# An assessment of the role of buttress roots in the carbon stocks of tropical forests

**DOI:** 10.3389/fpls.2025.1538583

**Published:** 2025-05-01

**Authors:** Xu Wang, Brian Njoroge Mwangi, Guangyi Zhou, Mengmeng Yang, Yuelin Li

**Affiliations:** ^1^ Tropical Forestry Research Institute, Chinese Academy of Forestry, Guangzhou, Guangdong, China; ^2^ Beijiangyuan National Forest Ecosystem Research Station, Guangzhou, China; ^3^ Key Laboratory of Vegetation Restoration and Management of Degraded Ecosystems, South China Botanical Garden, Chinese Academy of Sciences, Guangzhou, China; ^4^ Guangdong Provincial Key Laboratory of Applied Botany, South China Botanical Garden, Chinese Academy of Sciences, Guangzhou, China

**Keywords:** carbon accounting, tree biomass, forest management strategies, soil carbon content, carbon dynamics

## Abstract

**Introduction:**

Assessing carbon stocks in tropical forests is crucial for understanding their role in mitigating climate change. Researchers have previously underestimated key factors contributing to carbon dynamics in tropical forests. This study aims to address this knowledge gap.

**Methods:**

This study collected soil samples and made physical measurements of buttressed, control, and non-buttressed trees in a tropical forest from 2020 to 2022.

**Results:**

Our findings reveal that a significant proportion of trees (69.57%) had 3 to 5 buttress roots per tree. The total average biomass of the buttress roots and the above-ground portion of the trees with buttress roots was calculated to be 8.5 tonnes/ha for buttress roots and 44.04 tonnes/ha for above-ground biomass. The buttress root biomass accounted for 16.18% of the total tree biomass. It was observed that the presence of buttress roots was associated with a higher soil organic carbon content by an average of 20.8% in the upslope areas with buttress roots regardless of the season. Tree species with buttress roots had on average 20% higher organic carbon content. The upslope area of trees with buttress roots had lower soil temperature and higher soil moisture when compared to the other sectors measured in the study. Regardless of the season, the soil respiration rate in the areas without buttress roots and the control areas was higher than in those with buttress roots. The presence of buttress roots positively affected soil nutrient concentration throughout the study period.

**Discussion:**

This research shows that buttress roots play a crucial role in carbon storage. By integrating buttress roots into carbon accounting models, we can obtain more accurate estimates of carbon stock potential and develop more effective conservation and restoration strategies for tropical forests.

## 1 Introduction

In the context of global climate change, reducing carbon dioxide (CO_2_) emissions and enhancing biological carbon stocks are critical in mitigating global warming (Ma et al., 2022). Tropical rainforests, covering only 7% of worldwide land area (Malhi et al., 2002), play a vital role in carbon absorption through photosynthesis (Pan et al., 2024). These forests hold nearly 30% of global carbon stocks and net primary productivity making them exceedingly important in the global carbon budget (Townsend et al., 2011; Hubau et al., 2020). Notably, because of the important role that tropical forests play in the global carbon budget, there is a need for accurate carbon stock estimations in these ecosystems to understand the global carbon balance and advance initiatives to reduce CO_2_ emissions through forest management (Friedlingstein et al., 2022).

Forest carbon stocks comprise two components: above-ground and below-ground biomass (Yadav et al., 2022). Buttress roots are a common phenomenon of the above-ground biomass in most tropical forests (Alencar et al., 2023). Trees evolved to have buttress roots mainly because competition for resources meant that trees had to grow quickly to reach the sunlight at the canopy and therefore needed greater structural support during rapid vertical growth (Newbery et al., 2008; Zhiyuan et al., 2012). Besides supporting and enhancing trunk mechanical stability, buttress roots fulfill other crucial ecological functions within the entire ecosystem (Newbery et al., 2008; Zhiyuan et al., 2012). For instance, they enhance heterogeneity and regulate understory diversity in tropical rainforests (Tang et al., 2011). Pandey, 2011 found that soil organic carbon (SOC), total nitrogen (N), mineralized N, and soil particle size in buttress root zones of tropical rainforest in South Andaman Island, India, were 18%, 52%, 38%, and 13% higher, respectively, than in non-buttress root zones (Pandey et al., 2011). The influence of buttress roots on the surrounding topography results in higher accumulations of litter, surface soil nutrients, and water content near the upslope of buttress roots as compared to the downslope, establishing a persistent water gradient even during the dry season (Facelli and Pickett, 1991; Pandey et al., 2011; Yadav et al., 2022). Studies have shown that soil respiration tends to decrease with decreasing soil moisture during the drought period (Makita et al., 2018). However, there have been no studies that have adequately addressed the ecological roles of buttress roots in regulating SOC, soil respiration, and soil nutrient components in tropical forests in China.

Allometric regression models, typically utilizing parameters such as basal diameter (BD) or diameter at breast height (DBH), are commonly used to estimate total aboveground biomass, and enable the calculation of individual tree biomass (Ketterings et al., 2001; Piotto, 2008). However, in the case of timber inventory in tropical rainforests, the measurement position of tree stems with buttress roots is often determined based on the height of these roots. Trees with the highest point of buttress root attachment below 1.3 m are measured at 1.3 m for DBH, while trees with the highest point of buttress root attachment above 1.3 m are measured at 0.5m above the highest point of the buttress roots (Pandey et al., 2011). As a result, biomass of buttress roots is often overlooked in calculating above-ground tree biomass. Similarly, limited empirical studies exist on below-ground biomass due to the complex and time-consuming sampling process as well as the labor-intensive and costly nature of the research (Facelli and Pickett, 1991; Li et al., 2023). Estimations frequently rely on relationships with aboveground biomass, disregarding the biomass of buttress roots.

This study focuses on the often ignored component of aboveground biomass carbon (C) in tropical forests—buttress roots and how their presence or lack thereof influence SOC and soil respiration, with a particular focus on species-specific effects. By analyzing the biomass of buttress roots, soil organic carbon in buttress root zones, and soil respiration, we can address the question, what is the contribution of buttress roots to carbon stocks in tropical forests? The main study objective was to find out the effect of the presence or absence of buttress roots on key soil parameters such as SOC, soil respiration, and, soil nutrient components over a three-year study period (2020-2022). Additionally, this research provides theoretical support for understanding the carbon stock capacity of tropical rainforest ecosystems and global carbon accounting. This study aimed to measure and report on the effects of the presence or absence of buttress roots on soil moisture, soil respiration, and soil carbon. Based on previous studies showing that soil respiration decreases with declining soil moisture during drought periods (Makita et al., 2018), and considering the influence of buttress roots on soil nutrient and water gradients (Facelli and Pickett, 1991; Pandey et al., 2011), we hypothesized that the higher the soil nutrients and water content the higher the soil microbial activity and root respiration.

## 2 Materials and methods

### 2.1 Study site

**Figure 1:**
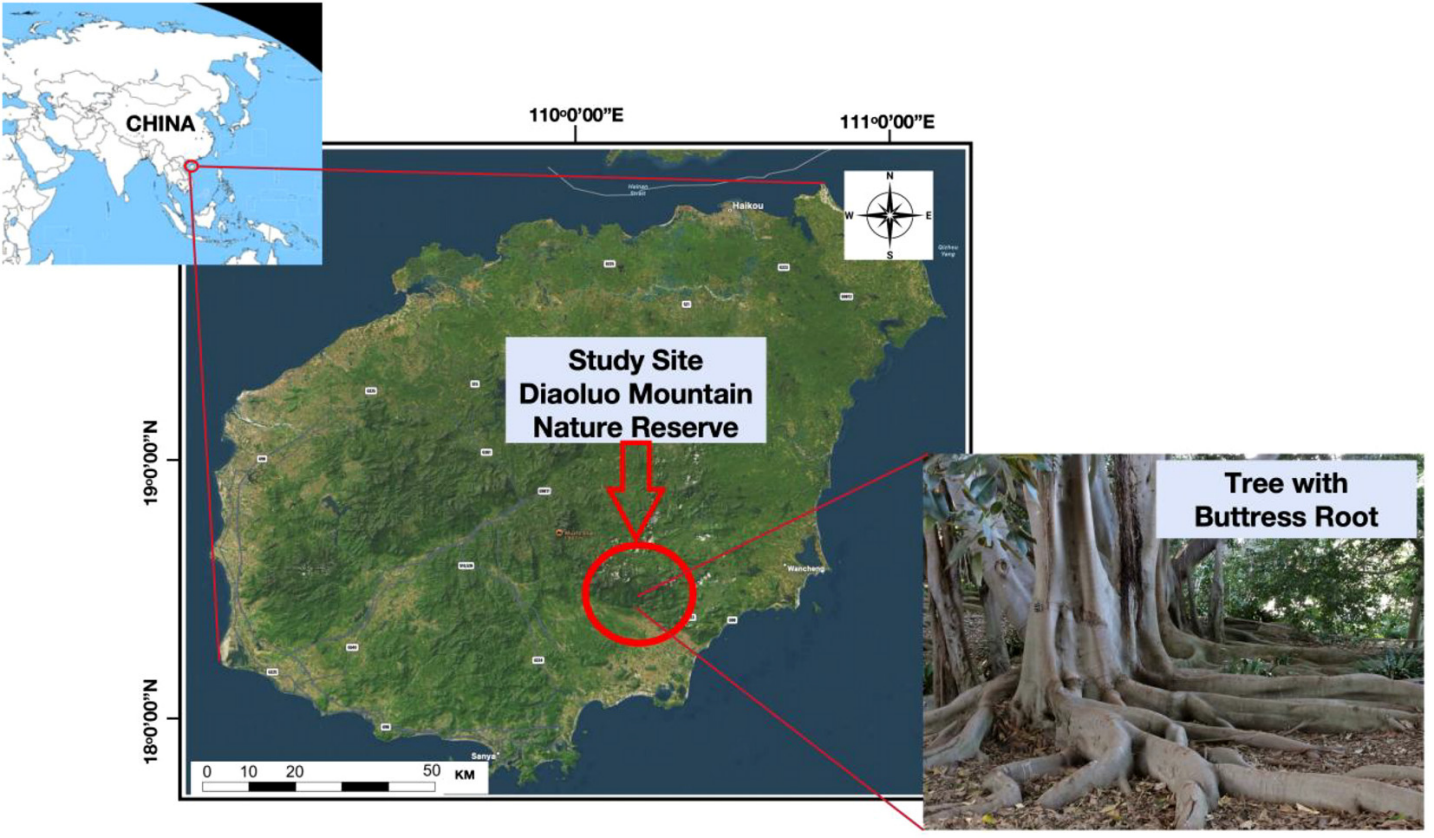
Study site location and a picture of a sampled tree.

The study site was situated within the pristine Diaoluo Mountain Nature Reserve located in Hainan Province, China (coordinates 18°43′-18°58′N, 109°45′-110°03′E) (**Figure 1**) (Wu et al., 2022). This area is renowned for its pristine tropical forest, 700 square kilometers in size, making it an important ecological site within China (Zhu et al., 2019).

The reserve had a tropical marine monsoon climate characterized by an average annual temperature of 24.6°C (Zhang et al., 2020). The warmest month, July, saw an average temperature of 28.4°C, while the coolest month, January, had an average temperature of 15.3°C, with the relative humidity consistently high at an average of 85.9% (Lin et al., 2014). The annual precipitation was 2160 mm, with a distinct division between the wet season from the end of May to October and the dry season from November to early May of the following year, with April serving as a transitional period between these two seasons (Wang et al., 2022) (**Table 1**).

**Table 1:** Study site features.

Diaoluo Mountain Nature Reserve	Site features
Location	18°43′-18°58′N, 109°45′-110°03′E
Size	700 km^2^
Climate	Tropical marine monsoon climate
Average annual temperature	24.6°C
Average relative humidity	85.9%
Average annual precipitation	2160 mm
Altitude range	100 to 1499 m

Topographically, the reserve comprises predominantly hilly terrain with elevations ranging from 100 to 1499 m above sea level, with the terrain being higher in the northern part and gradually descending towards the southern region (Yin et al., 2019). The main soil types in the study site were classified as ferralsoils according to the World Reference Base for Soil classification (WRB), comprising mainly mountainous lateritic red soil and mountainous yellow soil, which come from parent rocks such as granite and diorite, and are defined by their depth, moisture, acidity, lack of hardpans and organic matter content (Zhu et al., 2019).

The reserve’s vegetation features areas of pristine primary forests alongside extensive secondary forests. More than 3500 plant species have been identified in the reserve including 250 endangered orchids such as *Alsophila* sp*inulosa* and a rich under-storey of 15 *Dryopteris* and 12 *Diplopterygium* herbaceous genera (Zhu et al., 2019). Prominent tree species found within the reserve include *Vatica mangachapoi*, *Schima superba*, *Lithocarpus silvicolarum*, *Heritiera parvifolia*, and *Koilodepas hainanense* (Zhu et al., 2019).

### 2.2 Research methods

#### 2.2.1 Sample collection

**Figure 2:**
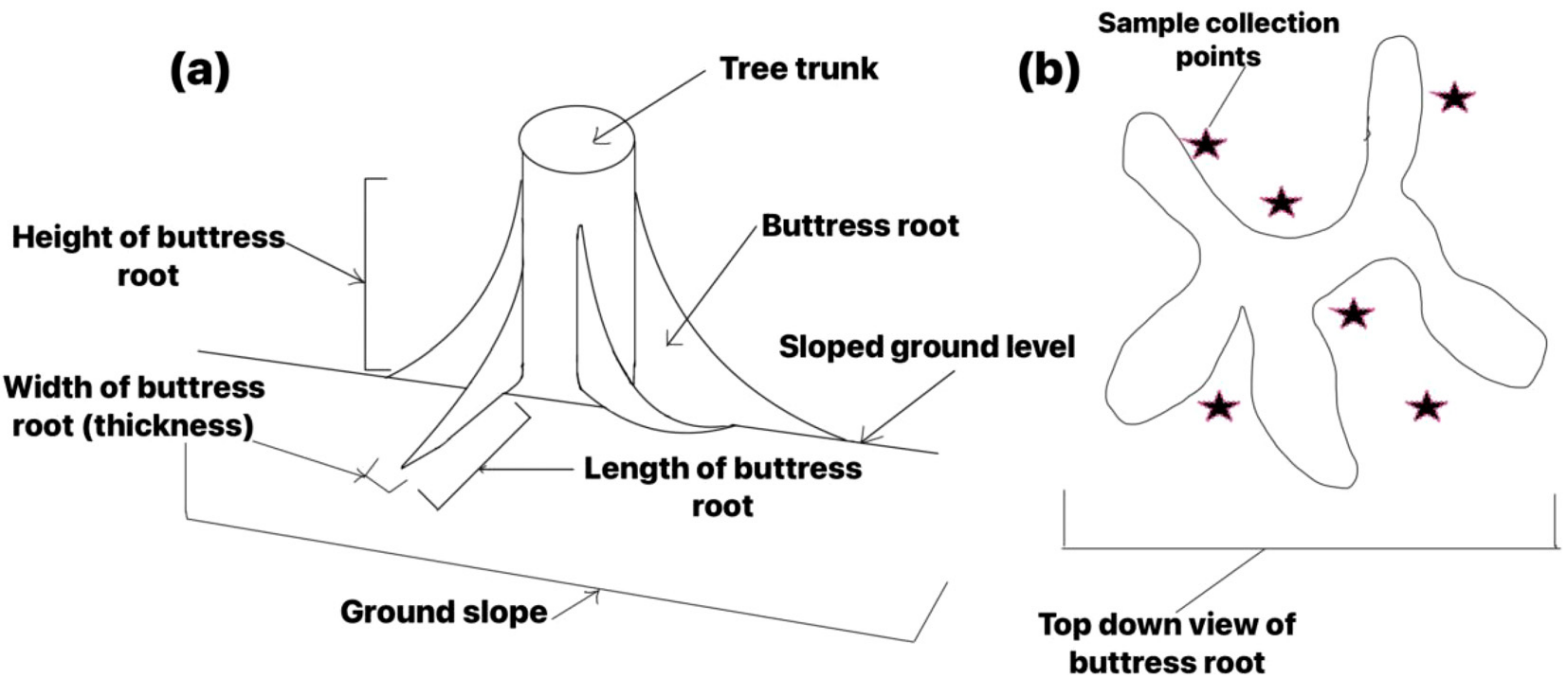
Schematic of soil sample collection points and buttress roots features **(a)** Cross-section of plate-rooted tree; **(b)** Plan view of plate-rooted tree).

Our study took place in the lowland rainforest region at an elevation of 300 m within the Diaoluo Mountain Nature Reserve, a 1-hectare (100 m by 100 m) vegetation plot was selected because it was situated in an undisturbed and representative forest area on a south facing aspect on a 15° slope gradient. This plot was further subdivided into 100 subplots, each measuring 10 m by 10 m. The 10 m by 10 m subplots were replicated along the landscape, and varied in topographic position namely topmost, middle, and downslope, to capture spatial variability. A Moran’s I parametric test was run in R to test that there was no spatial autocorrelation (RStudio Team, 2016). A buttress root was defined as a buttress-like projection of a tree root that extends from the trunk and is visible above the ground. The information recorded also included species identification, height, DBH, the presence of buttress roots, height at which buttress roots began, the number of buttress roots (each buttress protrusion from the stem), height and length of buttress roots, width of buttress roots at the proximal and distal ends of the tree trunk (**Figure 2**).

Within each of the 10 m x 10 m vegetation subplot, five trees with distinct buttress root structures (a length of more than 20 cm) and an average DBH of 28.5 ± 13.3 cm were selected as representatives of buttress-rooted trees, totalling 500 trees. For a matched-pair comparison analysis, five non-buttress-rooted trees with similar DBH and slope positions were chosen, for a total of 500 non-buttressed trees making up 5 buttressed trees and 5 non-buttressed trees per plot. Additionally, 3 trees randomized control trees were selected from each 10 m x 10 m subplot totalling 300 randomized control trees. Control trees served as a baseline for comparison, selected to match experimental trees in species, age, size, health, and environmental conditions, ensuring a fair evaluation of the effects of buttress roots on the studied variables. In total, this study sampled 1,300 trees. The main tree species found within the sampling area were *Symplocos poilanei*, *Schima crenata*, *Sapium discolor*, *Radermachera frondosa*, *Microcos paniculata*, *Pterospermum heterophyllum*, *Engelhardiarox burghiana*, *Castanopsis hainanensis*, *Amesiodendron chinense*, *Vatica mangachapoi*.

Soil sampling points were designated at various positions around the base of each tree trunk. These positions included 50 cm downslope of the tree trunk, 50 cm upslope of the tree trunk, as well as left relative to the upslope position and right relative to the upslope position (**Figure 2**). For each buttress-rooted tree and non-buttress-rooted tree, three soil sampling points were established at equal distances of 50 cm from the trunk in both the upslope and downslope directions.

Soil sample collection was conducted in August, corresponding to the rainy season, and in January, corresponding to dry season between 2020 and 2022 at two distinct soil layers: 0-10 cm and 10-30 cm depths. First, soil samples for measuring soil bulk density were collected from two trees per subplot at random at one sample point upslope and one sample point downslope of the tree (**Figure 2b**) using a soil corer (VSI SO Soil Corer 58 mm inside diameter), ensuring their original soil structure remained intact, and placed in labelled aluminium containers for subsequent determination of soil mechanical composition (texture and granulometric composition of soil) and soil density characteristics, these totalling 400 soil samples. Thereafter, a total of 18 (100 g) soil samples were collected from 3 representative trees in each sampling subplot during each sampling event every year (1 buttressed tree, 1 unbuttressed tree and 1 control tree), from 3 sample collection points upslope of the tree and 3 sample collection points downslope of the tree at 0-10 cm (50 g) and 10-30 cm (50 g) depths using a hand trowel as shown in **Figure 2**. This resulted in a total of 1,800 soil samples being acquired during each sampling period. These soil samples then underwent elimination of extraneous materials like gravel and plant debris. These portions from each sampling point (UB, DB, UUB, DUB and Control) were deposited in sealed plastic bags, labelled accordingly, air-dried and stored for further analysis of various soil physicochemical properties. These properties encompassed parameters such as pH, total carbon, total nitrogen, total phosphorus, total potassium, total hydrolysed nitrogen, total available potassium, and total available phosphorus, all of which were examined as explained in sections 2.2.3 and 2.2.4.

Subsequently, the soil samples were sieved through mesh sizes that were 0.25 mm, 0.15 mm, 0.075 mm, according to the specific analysis requirements outlined in section 2.2.3 and 2.2.4 (Li, 2019). Finally, the prepared samples were dispatched to the laboratory for an examination of soil physicochemical properties. The standard patented potassium dichromate oxidation external heating method (LY/T1237-1999) was used to find out how much organic matter was in the soil.

#### 2.2.2 Biomass calculation

This study calculated the buttress root volume and biomass. To calculate the buttress root volume, the height, the length and the width of the buttress roots (**Figure 2**) were measured. Thereafter, the formula in Equation 1 was applied to calculate the buttress root volume (Warren et al., 1988).

Buttress root volume calculation:


(1) $$\text{V}=0.5\left(\text{H}.\text{L}\right)\times 0.33\left(2{\text{W}}_{1}+{\text{W}}_{2}\right)$$

(Warren et al., 1988)


(2) $$\text{Biomas}{\text{s}}_{\text{E}}=\text{Densit}{\text{y}}_{\text{B}}+\text{Volume}\left(\text{V}\right)$$

(Li, 1993; Zhou et al., 2011)

*DensityB* was calculated as actual root weight (*WeightB*) divided by calculated root volume *(V)* (Equation 3). Root weight was measured from a small buttress root sample extracted from a tree a random from each subplot.


(3) $$Density_{B}=\frac{Weight_{B}}{Volume_{V}}$$

(Zhou et al., 2011)

Where *H* represents the height of buttress root (m) [measured longitudinally from the ground level to the termination point on the stem], *L* represents the length of buttress root (m) [the distance between the buttress root’s furthest point from the stem to the closest point to the stem measured transversely at the ground level], *W1* represents width of buttress root near the tree trunk (m) [measured transversely at the highest point from the ground where the buttress root attaches to the stem], and *W2* represents the width of buttress root away from the tree trunk (m) [measured transversely at the ground level] (Equation 1).

This research converted volume to biomass by multiplying the volume by the buttress root density as shown in Equation 2:

Where *BiomassE* is the buttress root biomass, *DensityB* is the buttress root density and Volume is the volume *(V)* as calculated in Equation 1.

#### 2.2.3 Determination of soil organic carbon components

The soil organic carbon was determined using the NaI heavy liquid fractionation method (Ola et al., 2019). Ten grams of air-dried soil samples, which had passed through a 2 mm sieve, were weighed into a 100 ml centrifuge tube. Subsequently, 40 ml of NaI solution with a density of 1.9 g/cm^3^ was added to the tube, and the mixture was oscillated for 60 minutes on a reciprocating shaker with a shaking speed of 250 times/min. The dispersed suspension was then centrifuged at 3000 rpm for 10 minutes. The suspended solids on the surface of the mixture were filtered through a 0.45μm microporous membrane to separate the light fraction organic matter. Following this, 20-30 ml of NaI solution was added to the centrifuge tube, and the same steps of separation, centrifugation, and collection of the reconstituted material were repeated (2-3 times). The collected reconstituted material was rinsed with a 0.01 mol/L CaCl_2_ solution and further rinsed with distilled water until no Cl^-^ reaction occurred. The reconstituted material was transferred to a pre-weighed 25 ml beaker, dried at 60°C for 24 hours, and then weighed to determine the proportion of reconstituted material (RMM). The organic carbon content was determined by grinding the material that passed through a 0.15 mm sieve and using the potassium dichromate oxidation-external heating method.

To calculate the reconstituted organic carbon content (ROC), Equation 4 was applied where ROC is the measured organic carbon (OC) multiplied by the reconstituted material mass (RMM) (Ola et al., 2019). To calculate “heavy fraction organic carbon” (HFOC), Equation 5 was applied where HFOC was calculated by multiplying total organic carbon (TOC) by dry soil mass (DSM) and the subtracting ROC obtained from Equation 4 (Ola et al., 2019).


(4) $$\text{ROC}\left(\frac{\text{g}}{\text{kg}}\right)=\text{OC}\times \text{RMM}$$

(Ola et al., 2019)


(5) $$\text{HFOC}\;\left(\frac{\text{gg}}{\text{kg}}\right)=\text{TOC}\;\times \text{DSM}-\text{ROC}$$

(Ola et al., 2019)

Where *ROC* is Reconstituted Organic Carbon; *OC* is Organic Carbon; *RMM* is Reconstituted Material Mass; *HFOC* is Heavy Fractional Organic Carbon; *TOC* is Total Organic Carbon Content; and *DSM* is Dry Soil Mass.

HFOC referred to a specific fraction of organic carbon in soil that was denser and typically more resistant to decomposition compared to lighter organic carbon fractions analyzed because it represents a more stable form of carbon that can be stored in soils for extended periods (Ola et al., 2019).

#### 2.2.4 Measurement of soil respiration

From the total 100 subplots, nine 10 x 10 m subplots were selected along the landscape with representative topographical positions namely along a gentle incline, 3 topmost subplots (1 buttressed tree, 1 unbuttressed tree, 1 control tree), 3 middle subplots (1 buttressed tree, 1 unbuttressed tree, 1 control tree), and 3 downmost subplots (1 buttressed tree, 1 unbuttressed tree, 1 control tree). Within each subplot, one representative healthy tree with an average DBH of 28.5 ± 13.3 cm was chosen and a PVC collar with an inner diameter of 20 cm and a height of 10 cm was installed at a distance of 80 cm from the tree trunk. The PVC soil collars were pressed into the soil at a depth of 8-10 cm, minimizing soil compaction caused by the PVC collars. The height of the PVC collar above the ground surface was approximately 2-3 cm. All aboveground parts of plants within the collars were completely removed, and the soil around the outer ring of the PVC collar was compacted to ensure no gas leakage. Within each of the nine subplots, soil respiration was measured using PVC collars installed at two points for buttressed trees (upslope and downslope) and one point each for unbuttressed and control trees, totaling 12 collars. Nine automated soil respiration modules were deployed, with each module sequentially measuring multiple collars, using the Li-8100A Soil CO_2_ Flux System (Li-COR Inc., Lincoln, NE, USA). Automated measurements were conducted during the rainy season (May to October) and the dry season (November to April). Soil moisture and soil temperature were assessed at depths of 5, 20, 40, 80, and 100 cm in each of the nine representative sampling subplots using 105-T and 107-L probes from Campbell Scientific Inc., as well as the CS616 probes from Campbell Inc., USA. Data logging was accomplished by employing CR10X (3) and CR23X (1) data loggers manufactured by Campbell Scientific Inc.

### 2.3 Statistical analysis

This study utilized the random effects model determined using the Hausman test in the ‘AER package ivreg’ package within R software (RStudio Team, 2016). The ‘ivreg package’ enabled the extension of measured numeric and graphical regression variables to linear models fitted by a two-stage least-squares (2SLS) regression. This enabled the modeling of correlations of data collected from the sampling plots in this study. *Post-hoc* LSD (Least Significant Difference) tests were performed to compare specific mean differences, with a significance level set at α = 0.05. The statistical analysis and data visualization were conducted using R software 3.6.3 in the ‘dplyr’ package (RStudio Team, 2016).

## 3 Results

### 3.1 Buttress root biomass

**Figure 3:**
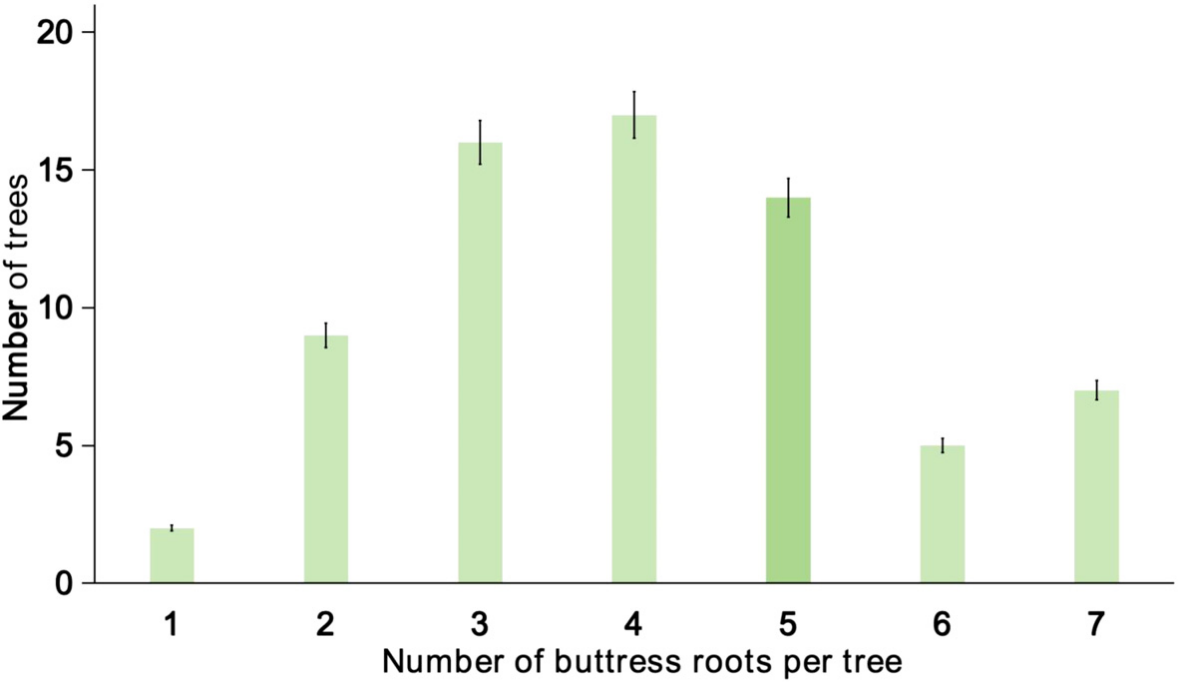
Distribution characteristics of the number of buttress roots per individual tree. Values are the mean of 300 replicates; error bars represent 95% confidence interval (LSD test).

In this study, a total of 1,300 trees with a diameter at breast height (DBH) greater than 10 cm were observed in the sample plots. Of the 500 buttress-rooted trees, the number of buttress roots ranged from 1 to 7 (**Figure 3**), with 69.57% having 3 to 5 roots per tree.

Using formulas (1) and (2), the total biomass of the buttress roots and the aboveground portion of the trees with buttress roots was calculated to be 8.5 tonnes/ha and 44.04 tonnes/ha, respectively. Buttress roots accounted for 16.18% of total tree biomass, with individual root biomass ranging from 2.3 to 3.8 tonnes/ha. Relative to total tree biomass, buttress roots contributed between 1.07% and 88.72%.

### 3.2 Impact of presence of buttress roots on soil organic carbon

**Figure 4:**
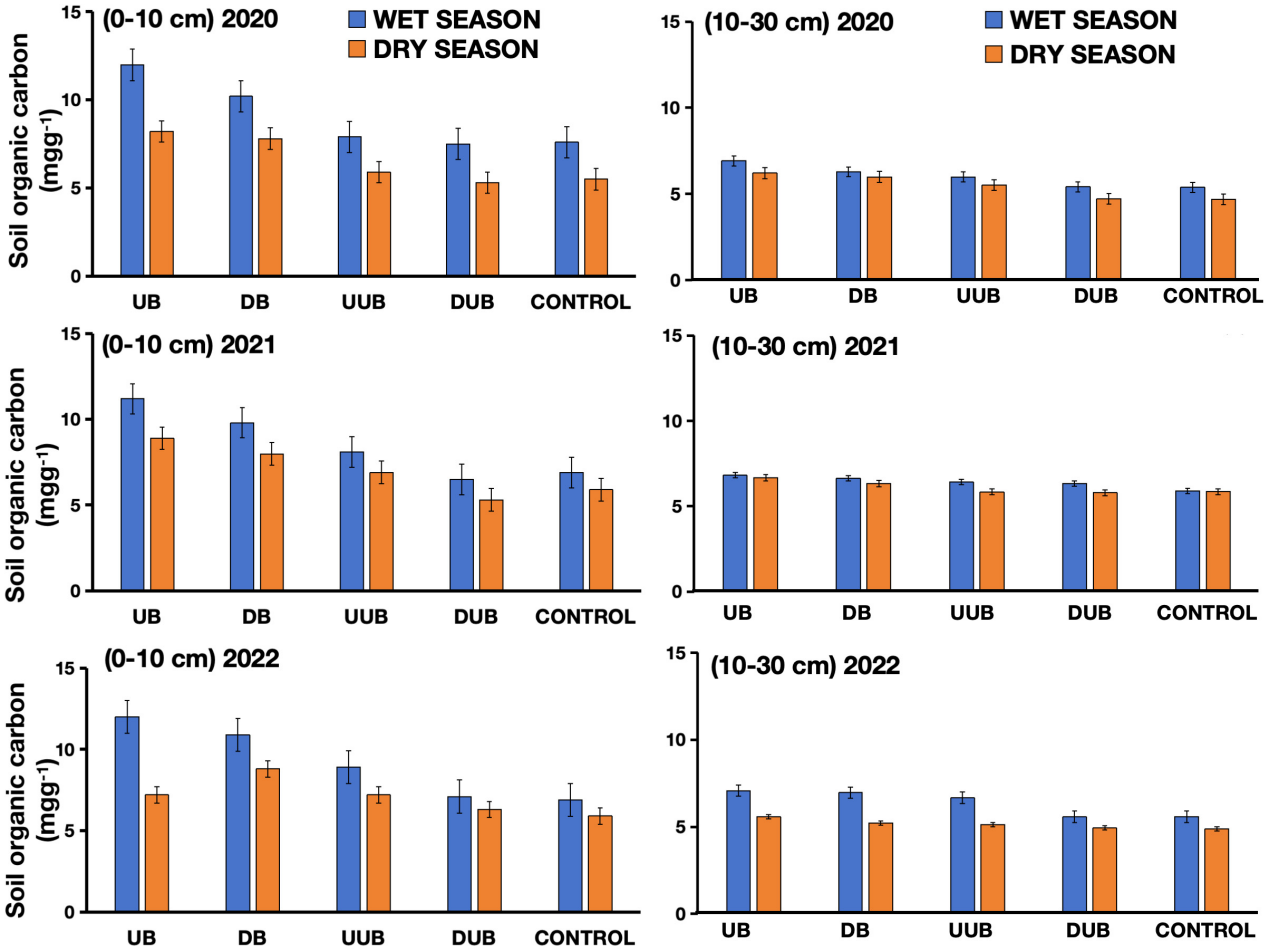
Comparison of soil organic carbon content between sectors with and without buttress roots in dry and wet seasons from 2020 to 2022. Soil layers are denoted as 1: 0-10 cm soil layer (on the left) and 2: 10-30 cm soil layer (on the right). Region labels include DB: down-slope of buttress roots area, UB, up-slope of buttress roots area; DUB, down-slope of unbuttressed roots area; UUB, up-slope of unbuttressed roots area; and CONTROL, control plot. Values are the mean of 300 replicates; error bars represent 95% confidence interval.

**Figure 5:**
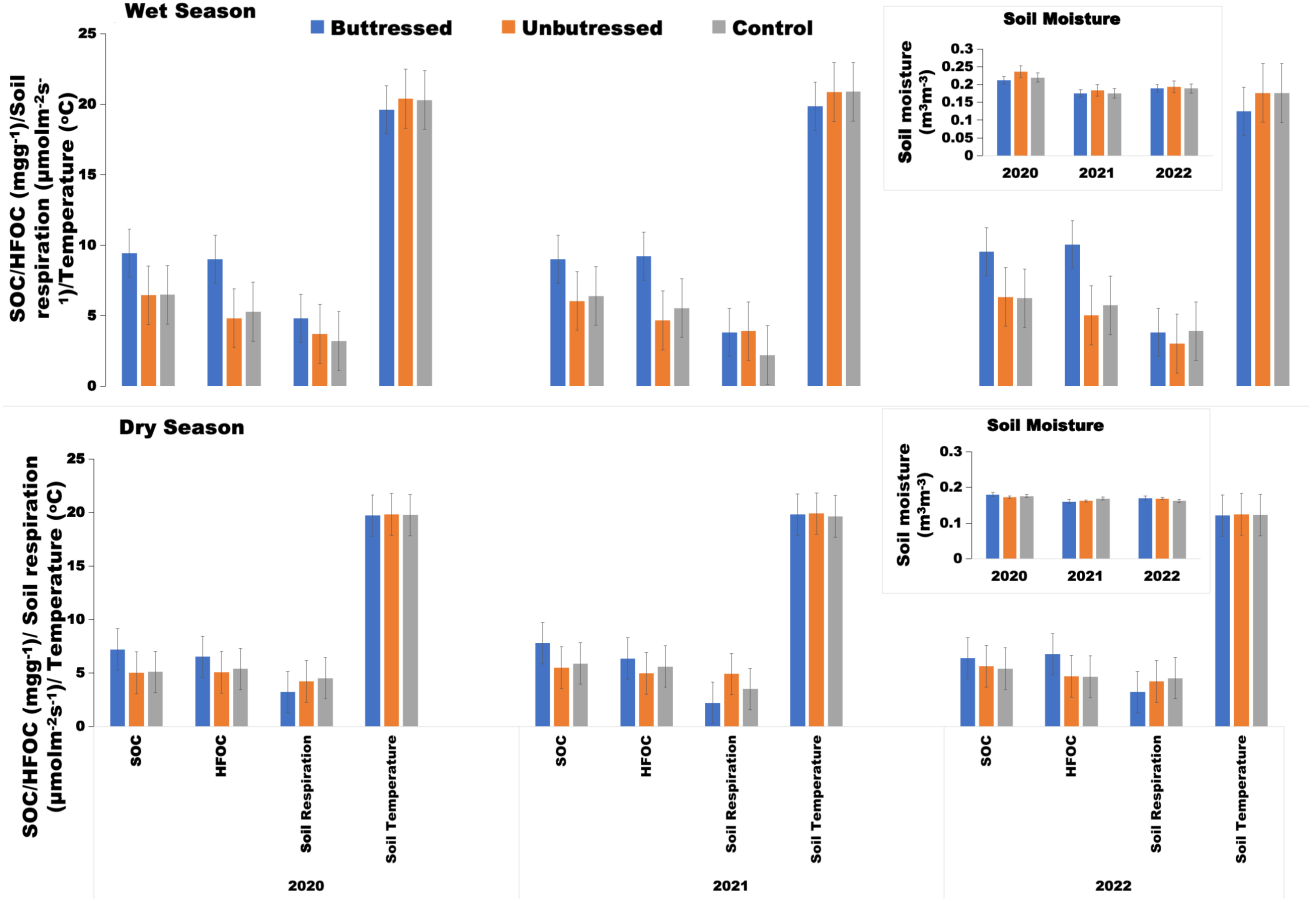
Changes in average soil moisture, average soil temperature, average soil respiration, average soil organic carbon (SOC), and average soil heavy fraction organic carbon (HFOC) recorded between 2020 and 2022 in sectors designated as Buttress Roots Area, Unbuttressed Roots Area, and Control during the wet season and the dry season. Values are the mean of 300 replicates; error bars represent 95% confidence interval.

Buttress roots consistently increased soil organic carbon in upslope areas compared to control and non-buttress groups over the three-year study period regardless of the season (**Figures 4**, **5**). During the rainy season, soil organic carbon declined by 5% between up-slope and down-slope areas with buttress roots and by 3% in both control and non-buttress areas in the 0-10 cm soil layer. Additionally, substantial differences were observed between the up-slope and down-slope areas with buttress roots and without buttress roots in the 10-30 cm soil layer vis-à-vis the control group. In the 0-10 cm soil layer, the 3-year average soil organic carbon content in the up-slope area with buttress roots was 11.948 mg/g, which was 16.34%, 31.95%, and 37.31% higher than the down-slope area with buttress roots, up-slope area without buttress roots, and down-slope area without buttress roots, respectively (**Figures 4a–c**).

The control group’s soil organic carbon content (9.65 mg/g) in the 0-10 cm layer was 20% lower than in up-slope areas with buttress roots. In the 10-30 cm soil layer, the average soil organic carbon content in the up-slope area with buttress roots was 11.356 mg/g, which was 25.86%, 21.16%, 19.82%, and 46.21% higher than the down-slope area with buttress roots, up-slope area without buttress roots, the control group area and down-slope area without buttress roots, respectively (**Figures 4d–f**).

During the dry season, significant differences in soil organic carbon were observed between up-slope and down-slope areas with buttress roots, control areas, and non-buttress areas in both soil layers (0-10 cm and 10-30 cm). In the 0-10 cm soil layer, the average 3-year soil organic carbon content in the up-slope area with buttress roots was 10.442 mg/g, which was 34.91%, 32.14%, 35.63%, and 37.72% higher than the down-slope area with buttress roots, the up-slope area without buttress roots, the control group area and the down-slope area without buttress roots, respectively (**Figures 4a–c**). In the 10-30 cm soil layer, the average 3-year, dry season soil organic carbon content in the up-slope area with buttress roots was 8.948 mg/g, which was 33.55%, 31.09%, and 54.99% higher than the down-slope area with buttress roots, up-slope area without buttress roots, and down-slope area without buttress roots, respectively (**Figures 4d–f**).

### 3.3 Impact of the presence of buttress roots on soil HFOC content

**Figure 6:**
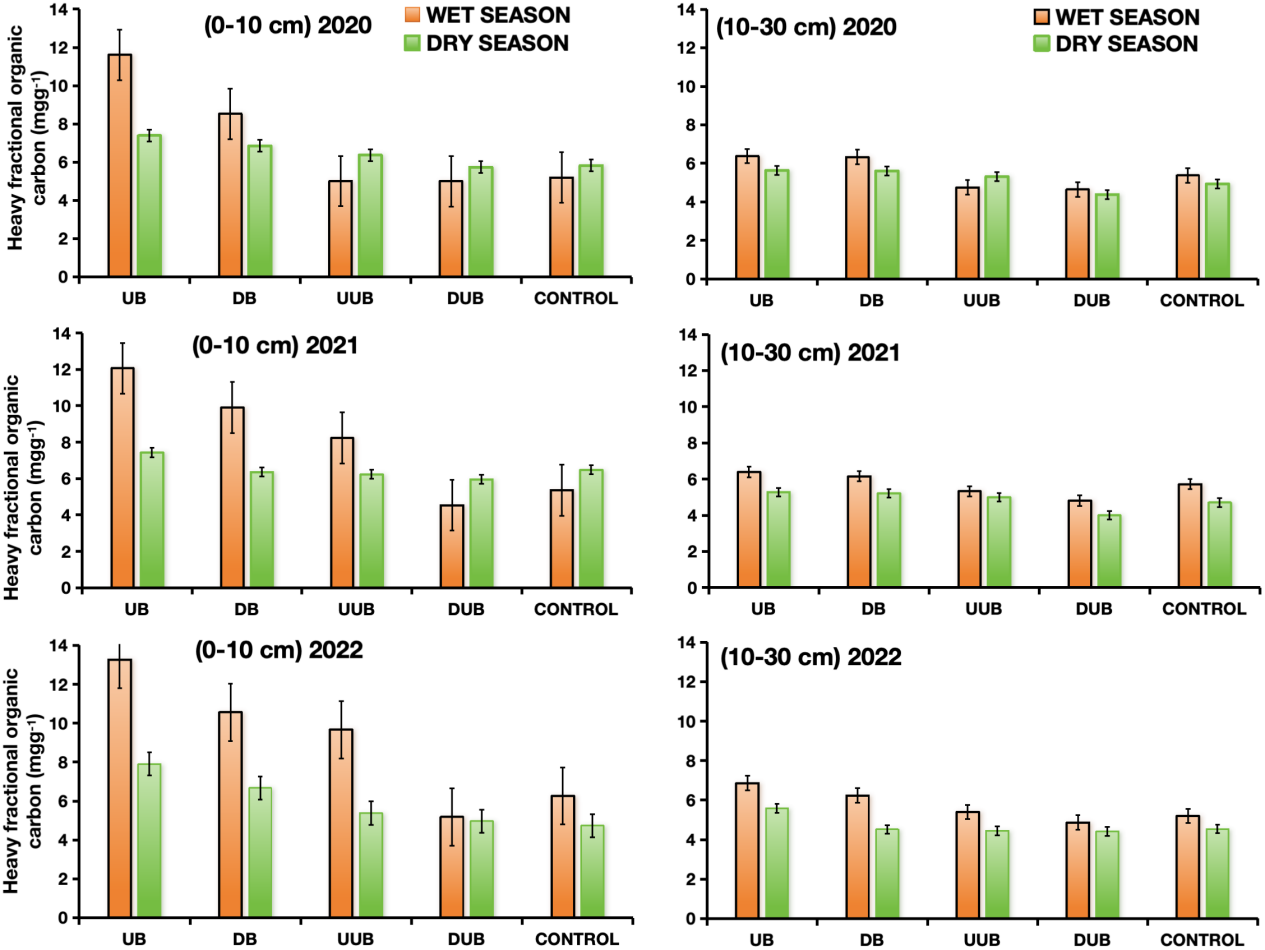
Variation in soil heavy fraction organic carbon content (HFOC) between sectors with and without buttress roots during the wet and dry seasons from 2020 to 2022. Soil layers are denoted as 1: 0-10 cm soil layer (on the left) and 2: 10-30 cm soil layer (on the right). Region labels include DB, down-slope of buttress roots area; UB, up-slope of buttress roots area; DUB, down-slope of unbuttressed roots area; UUB, up-slope of unbuttressed roots area; and CONTROL, control plot. Values are the mean of 300 replicates; error bars represent 95% confidence interval.

**Figure 6** compares the differences in soil HFOC content between areas with buttress roots, the control group area, and areas without buttress roots during the rainy and dry seasons from 2020 to 2022. HFOC in soil refers to the organic-inorganic composite carbon bound to soil mineral colloids. It represents the primary form of soil organic carbon and is an important indicator of soil carbon stock capacity. During the wet season, up-slope areas with buttress roots exhibited higher HFOC levels than down-slope areas, control areas, and non-buttress areas in both soil layers (**Figures 5**, **6**). In the 0-10 cm soil layer, the average content of HFOC in the up-slope area with buttress roots (12.126 mg/g) was higher by 31.05%, 29.15%, 31.55%, and 46.27% compared to the down-slope area with buttress roots, up-slope area without buttress roots, control group area, and down-slope area without buttress roots, respectively (**Figures 6a, c, e**). In the same wet season, a similar pattern was observed in the 10-30 cm soil layer though to a lesser statistical degree with buttress roots (6.27 mg/g) being higher by 9.5%, 10.25%, 12.63%, and 13.17% compared to the down-slope area with buttress roots, up-slope area without buttress roots, control group area, and down-slope area without buttress roots, respectively (**Figures 6b, d, f**). Additionally, during the dry season, in both the 0-10 cm and 10-30 cm soil layers, the up-slope areas with buttress roots exhibited higher levels of HFOC compared to the down-slope areas with buttress roots, the control group areas, and the up-slope and down-slope areas without buttress roots throughout the study period. In the 0-10 cm soil layer, the average content of HFOC in the up-slope area with buttress roots (10.962 mg/g) was higher by 32.01%, 30.25%, 32.65%, and 47.54% compared to the down-slope area with buttress roots, up-slope area without buttress roots, control group area, and down-slope area without buttress roots, respectively (**Figures 6a, c, e**). Similarly, in the 10-30 cm soil layer, the average content of HFOC in the up-slope area with buttress roots (9.518 mg/g) was higher by 28.22%, 25.12%, 32.83%, and 41.93% compared to the down-slope area with buttress roots, up-slope area without buttress roots, control area, and down-slope area without buttress roots, respectively (**Figures 6b, d, f**).

### 3.4 Impact of the presence of buttress roots on soil respiration

**Figure 7:**
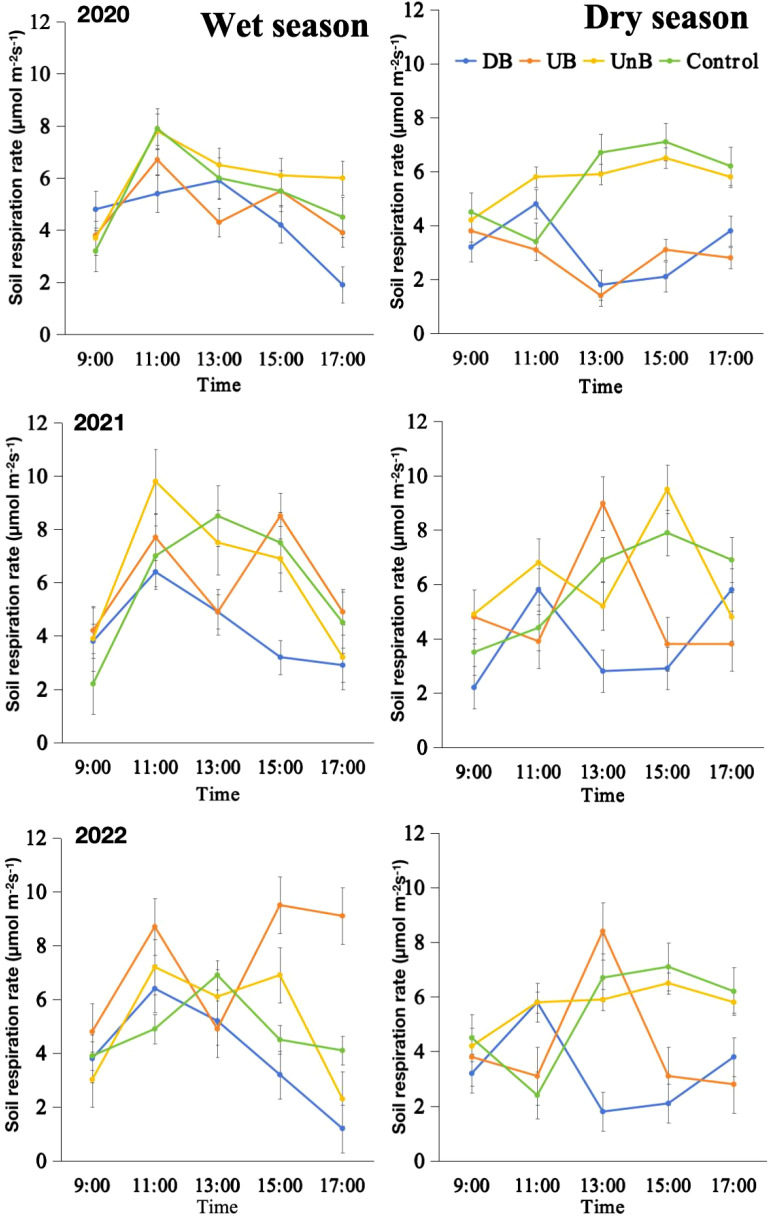
Average diurnal variation of soil respiration measured between measured between May to October (wet season; left column) and November to April (dry season; right column) in sectors with and without buttress roots (Rainy Season; Dry Season) between 2020 and 2022. Sectors are designated as follows: DB (Down-slope of Buttress Roots Area), UB (Up-slope of Buttress Roots Area), UnB (Unbuttress Roots Area both down-slope and up-slope) and Control is the control plot data. Values are the mean of of nine replicates (one per tree category per subplot); error bars represent 95% confidence interval (LSD test).

**Figure 7** illustrates the diurnal variation of soil respiration between areas with buttress roots, control group areas, and areas without buttress roots during the dry, and wet seasons between 2020 and 2022. Soil respiration is the main pathway through which CO_2_ is released from the soil to the atmosphere. Diurnal soil respiration patterns varied between up-slope and down-slope areas with buttress roots, control areas, and non-buttress areas (**Figure 7**). Soil respiration rates were consistently higher in non-buttress areas compared to buttress root and control areas across seasons (**Figures 5**, **7a, b**).

During the rainy season (**Figure 7a**), both the areas with buttress roots, the control group areas, and areas without buttress roots exhibited a similar pattern of increasing and then decreasing soil respiration, with the peak occurring around 11:00. However, the peak of soil respiration in areas with buttress roots occurs slightly later, around 13:00.

During the dry season (**Figures 5**, **7b**), the variation patterns of soil respiration differed among the up-slope and down-slope areas of the buttress roots, the control group areas, and the areas without buttress roots. In the up-slope area with buttress roots, soil respiration showed an increasing trend followed by a decrease and then another increase, with peaks occurring around 11:00 and 17:00. In the downslope area with buttress roots, soil respiration generally exhibits a decreasing trend. In the areas without buttress roots, soil respiration shows an initial increase followed by a decrease, with the peak occurring around 15:00. The control group respiration exhibits a similar pattern to the areas without buttress roots.

### 3.5 Impact of the presence of buttress roots on various soil nutrient components

Analysis of 2020-2022 data revealed significant correlations (P<0.05) among primary soil chemical indicators, excluding phosphorus, in the dry season (**Table 2**). The highest correlation coefficient (r=0.728) was between soil organic carbon and nitrogen (**Table 2**). This was a statistically higher correlation when compared to the control group and the areas without buttress roots. This is followed by the correlation between organic carbon and potassium (r=0.305), nitrogen and potassium (r=0.298), nitrogen and available phosphorus (r=0.260), and phosphorus and potassium (r=0.236). All of these correlations demonstrate a positive relationship. This positive correlation was also observed in the control group area and in areas without buttress roots although to a lesser degree (**Table 3**). A tenuous positive association exists between the overall potassium content in soil and other physicochemical indices. However, there is no correlation (P>0.05) between soil-available potassium and phosphorus or nitrogen. A significantly weak negative association (P<0.05) exists between soil pH and the levels of organic carbon and nitrogen. There was no significant relationship between phosphorus and other soil physicochemical indices (P>0.05). Overall, the presence of buttress roots positively affected soil nutrient concentration in the dry season compared to the control group areas and areas without buttress roots (**Tables 2**, **3**).

**Table 2:** The analysis of the averages (2020-2022) of the main chemical soil properties of samples taken from trees with buttress roots in the dry season.

Index	Organic carbon (g/kg)	Nitrogen (g/kg)	Phosphorus (g/kg)	Potassium (g/kg)	pH
Organic carbon (g/kg)	1.000				
Nitrogen (g/kg)	0.728*	1.000			
Phosphorus (g/kg)	0.059	0.260	1.000		
Potassium (g/kg)	0.305*	0.298*	0.236	1.000	
pH	-0.192*	-0.177*	0.206	0.076	1.000

A p-value of less than 0.05 was flagged with one star (*).

**Table 3:** The analysis of the averages (2020-2022) of the main chemical soil properties of samples taken from trees without buttress roots and the control group in brackets, in the dry season.

Index	Organic carbon (g/kg)	Nitrogen (g/kg)	Phosphorus (g/kg)	Potassium (g/kg)	pH
Organic carbon (g/kg)	1.000 (1.00)				
Nitrogen (g/kg)	0.452* (0.413)	1.000 (1.000)			
Phosphorus (g/kg)	0.012 (0.014)	0.12 (0.101)	1.000 (1.000)		
Potassium (g/kg)	0.135* (0.102)	0.097* (0.093)	0.057 (0.053)	1.000 (1.000)	
pH	-0.046* (-0.034)	-0.062* (-0.051)	0.354 (0.351)	0.021 (0.012)	1.000 (1.000)

A p-value of less than 0.05 was flagged with one star (*).

From the analysis of the mean values of various soil nutrients measured between 2020 to 2022, **Table 4** reveals a minor alteration in the association among several indicators during the wet season. In addition to soil pH, there is a strong positive association (P<0.05) between the physical and chemical indicators of the soil in the root area during the wet season. The correlation coefficient between nitrogen and accessible phosphorus is the highest (r=0.919), followed by the correlation between organic carbon and nitrogen (r=0.897), and the correlation between organic carbon and phosphorus (r=0.786) (**Table 4**). This positive correlation was also observed in the control group area and areas without buttress roots, although to a lesser degree (**Table 5**). The relationship between soil pH and other physicochemical properties is not strong. There is a significant positive correlation between soil pH and nitrogen (P<0.05). On the other hand, there is a significant negative correlation between soil pH and potassium (P<0.05). Overall, soil organic carbon was observed to be influenced by soil chemical properties, with soil nitrogen having the highest statistical influence, followed by phosphorus and potassium both during the dry and wet seasons. Buttress roots positively influenced soil nutrient concentrations in the dry season compared to control and non-buttress areas (**Tables 4**, **5**).

**Table 4:** The analysis of the averages (2020-2022) of the main chemical soil properties of samples taken from trees with buttress roots in the wet season.

Index	Organic carbon (g/kg)	Nitrogen (g/kg)	Phosphorus (g/kg)	Potassium (g/kg)	pH
Organic carbon (g/kg)	1.000				
Nitrogen (g/kg)	0.897*	1.000			
Phosphorus (g/kg)	0.786*	0.919*	1.000		
Potassium (g/kg)	0.291*	0.542*	0.617*	1.000	
pH	0.028	0.219	-0.054	-0.382*	1.000

A p-value of less than 0.05 was flagged with one star (*).

**Table 5:** The analysis of the averages (2020-2022) of the main chemical soil properties of samples taken from trees without buttress roots in the wet season.

Index	Organic carbon (g/kg)	Nitrogen (g/kg)	Phosphorus (g/kg)	Potassium (g/kg)	pH
Organic carbon (g/kg)	1.000 (1.000)				
Nitrogen (g/kg)	0.586* (0.5652)	1.000 (1.000)			
Phosphorus (g/kg)	0.421* (0.4102)	0.512* (0.5013)	1.000 (1.000)		
Potassium (g/kg)	0.101* (0.1122)	0.202* (0.2153)	0.126* (0.1145)	1.000 (1.000)	
pH	0.002 (0.0012)	0.019 (0.0265)	-0.021 (-0.0241)	-0.152* (-0.1342)	1.000 (1.000)

A p-value of less than 0.05 was flagged with one star (*).

### 3.6 The impact of buttress roots on soil organic carbon in different tree species

**Figure 8:**
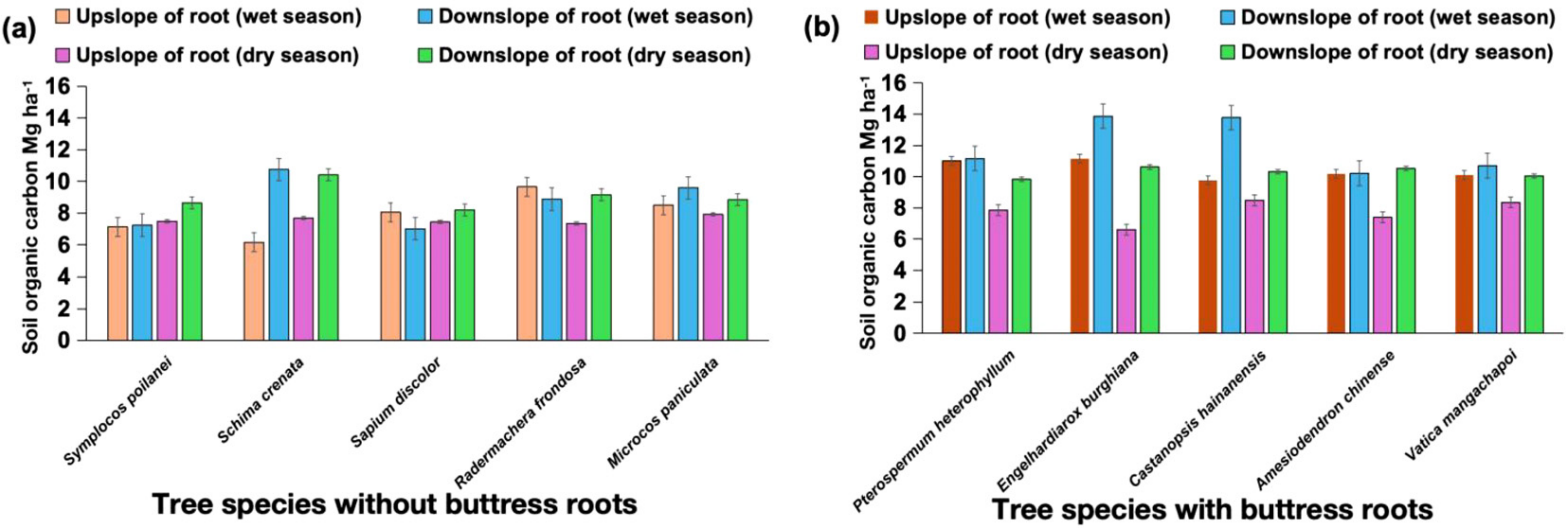
Average soil organic carbon in different tree species **(a)** without buttress roots and **(b)** with buttress roots measured from 2020 to 2022. Values are the mean of 200 replicates; error bars represent 95% confidence interval.

Tree species with buttress roots had 20% higher organic carbon content on average during both wet and dry seasons (**Figure 8**). Among the trees without buttress roots, the tree species *Schima crenata* had the highest average soil organic carbon (11.2 Mg ha^-1^) during the wet season compared to the lowest average which was *Symplocos poilanei* (7.2 Mg ha^-1^) during the wet season. Buttressed, unbuttressed, and control group trees had significantly lower soil organic carbon content (25% lower) on average during the dry season compared to the wet seasons of the study period (2020 to 2022). Among the trees with buttress roots, *Engelhardiarox burghiana* had the highest average organic carbon content (14.1 Mg ha^-1^) compared to *Castanopsis hainanensis* which has the lowest average (9.2 Mg ha^-1^) during the wet season.

### 3.7 The impact of buttress roots

**Figure 9:**
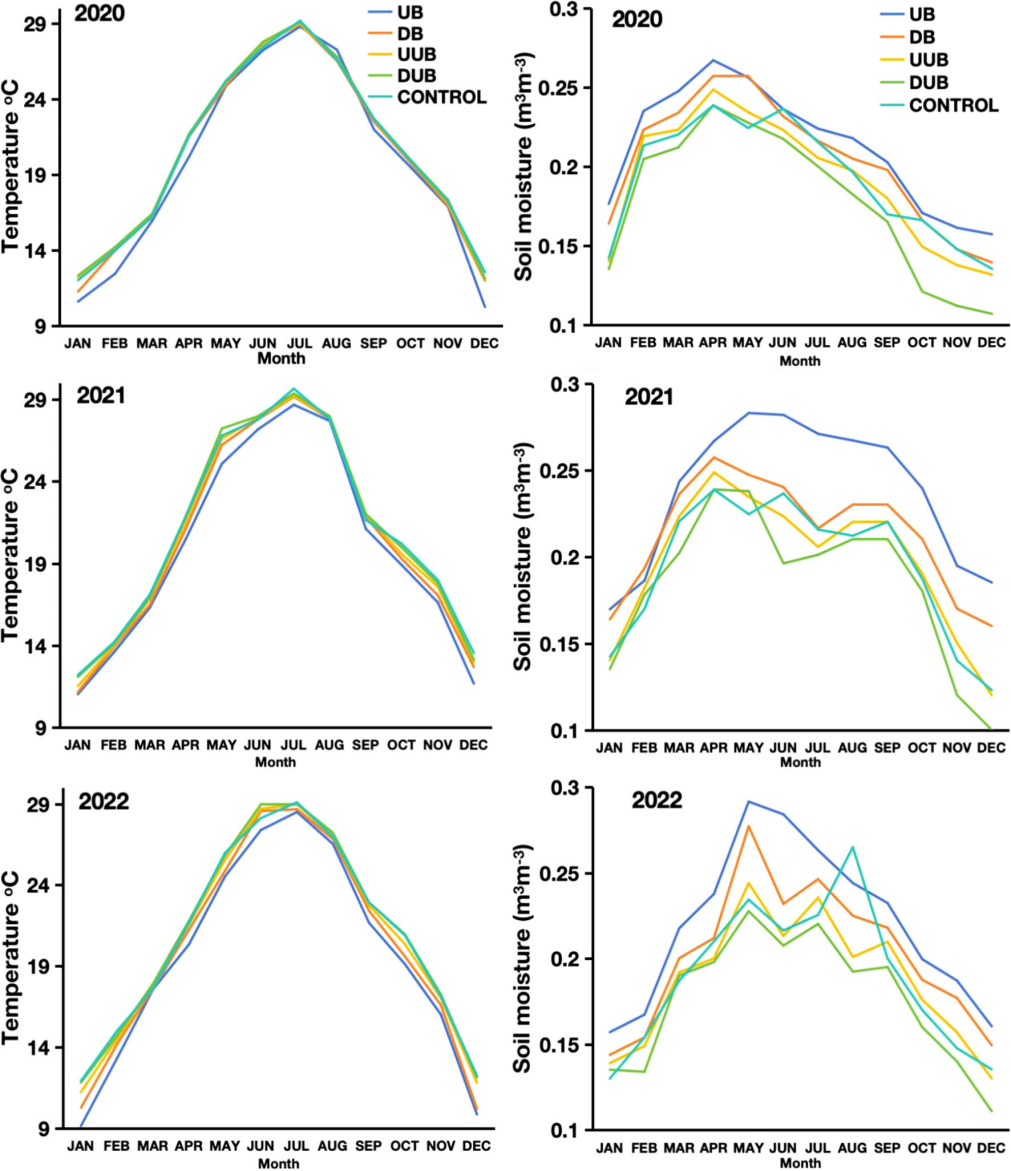
The seasonal changes in average soil temperature (left) and average soil moisture (right) from 2020 to 2022 in sectors designated as follows: DB (Downslope of Buttress Roots Area), UB (Upslope of Buttress Roots Area), UnB (Unbuttress Roots Area both downslope and upslope), and Control is the control plot data.

From the analysis in **Figure 9**, the presence of buttress roots specifically in the up-slope area corresponded to a decrease in soil temperature by an average of 0.5 ± 0.1 °C when the soil temperature measured over a three-year study period (2020-2022) was compared against the down-slope area with buttress roots, upslope area without buttress roots, control group area and down-slope area without buttress roots (**Figures 5**, **9a**). Soil moisture was higher on the up-slope area of trees with buttress roots by an average 10% (0.16 m^3^m^-3^) when compared to the downslope area with buttress roots, upslope area without buttress roots, control group area, and downslope area without buttress roots (**Figures 5**, **9b**). The measurements of soil temperature and soil moisture exhibited a season pattern with the wet season having higher soil temperature and higher soil moisture while the dry season had lower soil moisture and lower soil temperature. Increased soil moisture consistently corresponded to lower soil temperatures across all seasons. Soil temperature and soil moisture showed an inversely proportional relationship with the results showing that an increase in soil moisture correlated to a decrease in soil temperature (y=0.3546x+20.6542 R^2^ = 0.76 p<0.001). The upslope area of trees with buttress roots was therefore cooler and wetter when compared to the other sectors measured in the study.

Buttress roots increased soil organic carbon content in both seasons compared to non-buttress and control areas (**Figure 5**). Additionally, the presence of buttress roots correlated with higher levels of heavy fraction organic carbon compared to sample areas without buttress roots and the control group areas (**Figure 5**). Moreover, regardless of the season, the soil respiration rate in the areas without buttress roots was higher than that in the areas with buttress roots and the control group areas. Soil moisture was higher in sample areas with buttress roots compared to the areas without buttress roots, and control group areas (**Figure 5**).

### 3.8 Statistical relationship between measured soil moisture, and soil temperature against the magnitudes of the measured SOC, soil heavy fraction organic carbon, and soil respiration

**Figure 10:**
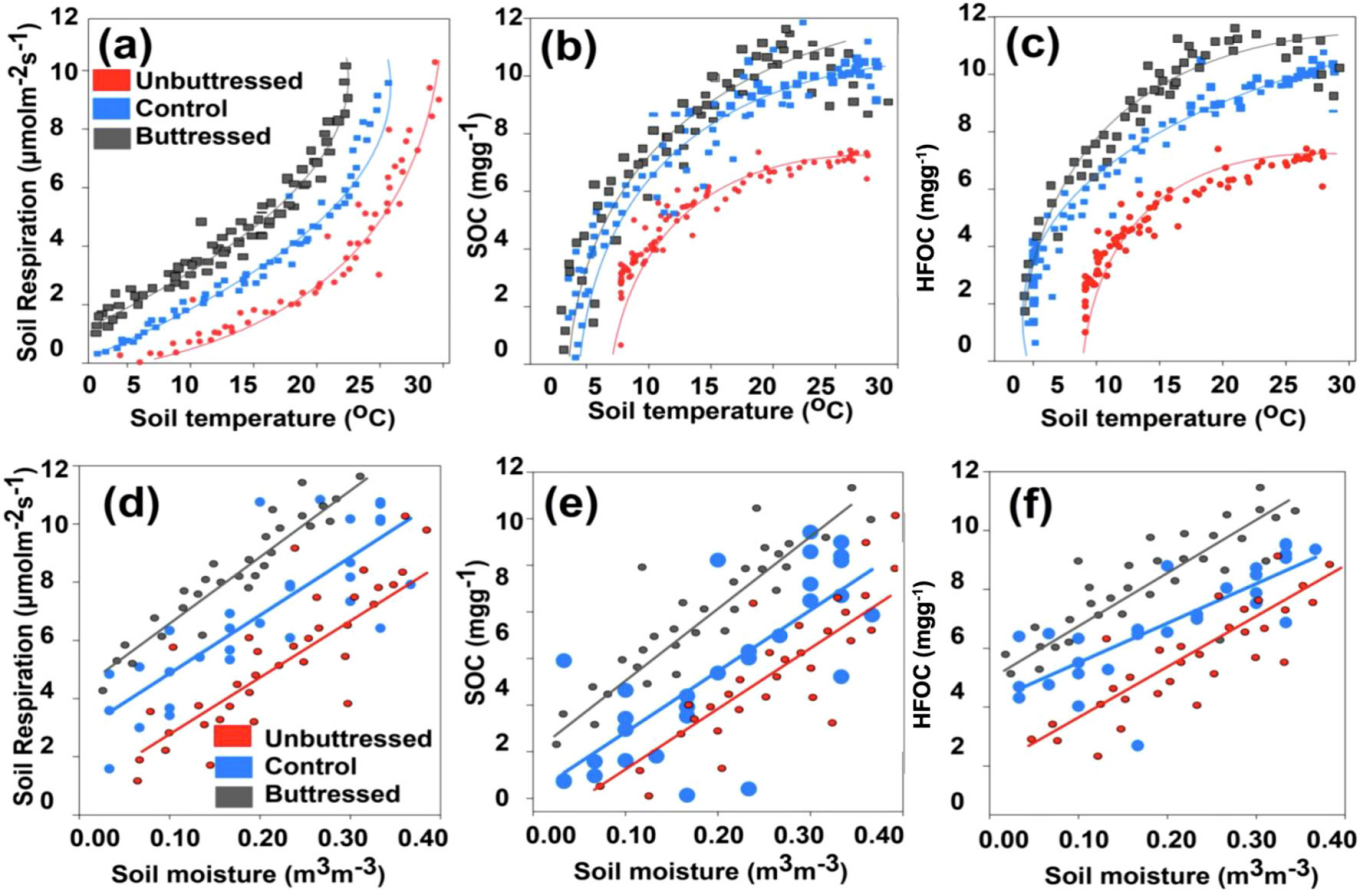
The statistical relationship between average soil moisture **(a–c)** and soil temperature **(d–f)** against the magnitudes of the average SOC, soil heavy fraction organic carbon (HFOC), and soil respiration recorded between 2020 and 2022 in areas designated as Buttress Roots Area, Unbuttressed Roots Area, and Control.

Temperature significantly influenced soil respiration, SOC, and heavy fraction organic carbon, showing exponential increases (R^2^ = 0.83, 0.78, 0.87; p<0.0001) up to a threshold, beyond which values declined (**Figures 10a–c**). In **Figures 5** and **9**, it was shown that temperature was lower in areas with buttress roots compared to unbuttressed areas. Temperature was found to be a key variable that affected soil respiration, SOC, and soil heavy fraction organic carbon both in the wet and dry seasons, and the statistical relationship followed a similar pattern in the unbuttressed, control, and buttressed sample points.

The relationship between soil moisture, soil respiration, SOC, and soil heavy fraction organic carbon was significant and had a linear relationship with an increase in soil moisture resulting in a corresponding increase in soil respiration (R^2^ = 0.78, p<0.001), SOC (R^2^ = 0.79, p<0.001) and soil heavy fraction organic carbon (R^2^ = 0.81, p<0.001) in the buttress root and unbuttressed sample plots (**Figures 10d–f**). In **Figures 5** and **9**, it was shown that soil moisture was higher in areas with buttress roots compared to unbuttressed areas. Soil moisture was found to be a key variable that affected soil respiration, SOC, and soil heavy fraction organic carbon both in the wet and dry seasons, and the relationship followed a similar pattern in the unbuttressed, control, and buttressed sample points.

## 4 Discussion

### 4.1 The effect of the presence of buttress roots on soil carbon content

The results of this study reaffirm the important role of buttress roots in influencing soil carbon content within tropical forests, aligning with previous research findings (Dean et al., 2020). Previous studies have often overlooked the contribution of buttress roots to biomass, a key indicator of forest carbon stock capacity, due to their unique and irregular structure. However, our findings indicate that buttress roots account for 44.27% of the total biomass in plate-rooted trees. The individual variation in buttress root biomass ranged from 2.3 tonnes/ha to 3.8 tonnes/ha contributing between 1.07% and 42.87% to the total tree biomass. Recognizing the biomass of buttress roots is crucial for assessing tropical forest carbon stocks, as plate-rooted trees constitute 12% to 35% of these ecosystems (Chapman et al., 1998; Milliken, 1998). Plate-rooted trees typically have tall trunks and canopies, with their plate roots elongating as trees age and increase in diameter at breast height (Woodcock, 2000; Warner et al., 2017).

The advent of three-dimensional laser scanning technology offers a promising avenue for further studying buttress roots (Næsset and Gobakken, 2008). Due to the challenges of measuring underground biomass, estimates often rely on above-ground biomass data (Kenzo et al., 2020).

### 4.2 The influence of the presence of buttress roots on soil organic carbon

Buttress roots play a multifaceted role in shaping soil organic carbon dynamics within tropical forests (Ola et al., 2019). Our findings underscore the pivotal role of buttress roots in affecting soil organic carbon levels and distribution within the ecosystem. The presence of buttress roots creates ‘root walls’ (Herwitz, 1988), which impede down-slope material flow. This reduction in surface runoff and erosion caused by rainfall creates unique ground biogeochemical zones (Pandey et al., 2011). The observed increase in soil organic carbon content in areas with buttress roots can be attributed to several factors. First, buttress roots contribute to the accumulation of organic matter derived from their own structure and their presence fosters enhanced nutrient cycling (Pandey et al., 2011). Buttress roots provide extensive surface area, stabilize tree trunks, and create microenvironments conducive to organic matter accumulation and nutrient-rich soil formation (Epron et al., 2025).

Leaf litter represents a primary source of soil organic carbon. An increase in leaf litter quantity in plate-rooted areas, as indicated by Pandey, may contribute to the elevated soil organic carbon content in these sectors (Pandey et al., 2011). Some studies have shown that an increase in leaf litter quantity over 15 consecutive years, led to a doubling of soil carbon storage in tropical forests (Sayer et al., 2019).

Soil organisms and microorganisms play a crucial role in soil organic carbon dynamics by decomposing leaf litter (Strecker et al., 2021). Buttress-rooted areas exhibit higher leaf litter quantities compared to non-buttress-rooted areas, leading to increased species diversity and abundance of soil animals. These conditions enhance biogeochemical cycling, potentially contributing to higher soil organic carbon levels, particularly in upper slope positions.

The influence of buttress roots extends beyond soil organic carbon content to encompass other soil properties, including moisture retention, nutrient availability, and soil structure (Zürcher, 2021). The elevated soil moisture near buttress roots supports plant growth and enhances overall ecosystem productivity. Alterations in soil structure and nutrient distribution, induced by the presence of buttress roots, contribute to the ecological functioning of the forest ecosystem, as our results show.

Additionally, higher precipitation was found to result in higher soil organic carbon in the non-buttress root trees sampled, both in the DUB and UUB sectors (**Figure 8**). These findings align with studies in other ecosystems, where the wet season showed elevated levels of phenolics, SOC, and iron oxides compared to the dry season (Davidson et al., 2006). In this study, SOC significantly increased with increasing soil moisture and temperature (**Figure 10**). The presence of buttress roots reduces soil temperature and increases soil moisture (**Figure 9**), influencing total SOC (**Figure 4**).

### 4.3 The impact of the presence of buttress roots on soil respiration

Soil respiration, a critical pathway for releasing CO_2_ from the soil to the atmosphere, exhibits varying diurnal patterns in areas with and without buttress roots (Nottingham et al., 2021). Our findings indicate that, overall, soil respiration rates in areas without buttress roots surpass those in areas with buttress roots during both the rainy and dry seasons. These differences in soil respiration patterns arise from multiple factors.

One important factor is precipitation. During the rainy season, both areas with and without buttress roots display similar patterns of increasing and decreasing soil respiration. In contrast, during the dry season, the variation in soil respiration differs in key ways namely; in the upslope area with buttress roots, soil respiration shows an increasing trend, followed by a decrease and another increase while, in the downslope area with buttress roots, soil respiration generally exhibits a decreasing trend. In areas without buttress roots, soil respiration follows an initial increase, followed by a decrease. These are similar patterns to what has been found by other researchers (Davidson et al., 2006; Makita et al., 2018; Nottingham et al., 2021).

Multiple factors can contribute to the lower soil respiration rates observed in areas with buttress roots. Soil respiration is mostly caused by tree roots and microbes. The Plate Root Nutrient Hypothesis says that plate roots are designed to take in nutrients and water efficiently to deal with soil that is low in nutrients (Black and Harper, 1979). The enhanced nutrient uptake capacity of plate roots may result in a reduced overall quantity of roots in the soil, including root respiration.

This study analyzed soil moisture and temperature, finding decreased temperature and increased moisture in areas with buttress roots. Alongside this finding, uphill sectors of buttress root trees had the highest soil temperature and the lowest soil temperature, thus affecting soil respiration and other soil parameters in this area. Soil respiration was significantly correlated to soil moisture and soil temperature with an increase in soil moisture and soil temperature increasing soil respiration (**Figure 10**). Because the presence of buttress roots results in a reduction in soil temperature (**Figure 9**) and an increase in soil moisture (**Figure 9**), the study deduced that variations in two of these key variables affected the total soil respiration in a statistically quantifiable manner (**Figures 7**, **5**, **10**). Research by Tang et al, 2011 in the tropical seasonal rainforest slope in Xishuangbanna, Southwest China, recorded a similar effect of buttress roots on soil moisture and soil temperature.

### 4.4 Impact of the presence of buttress roots on various soil nutrient components

The chemical characteristics of the soil have a direct impact on the development of plants. Analysis of soil chemical properties revealed significant differences between the upper and lower slopes of the buttress root zone. Furthermore, the nutrient gradient between the slopes of the buttress root zone was found to be higher compared to the non-buttress root zone. This suggests that a zone of increased soil nutrient enrichment had developed along the slope gradient of the buttress root zone, leading to an increase in the amount of soil organic carbon and consequently enhancing the variability of the soil in the root zone. This finding further validates that buttress roots enhance soil heterogeneity (Pandey et al., 2011). This study detected variations in pH levels between the higher and lower slopes of the buttress root zone, as well as across distinct soil layers, throughout both the dry and rainy seasons. During the dry season, the pH value of the soil in the 0-10 cm layer where the buttress roots are located was notably higher compared to the non-buttress root zone. This finding contradicts the research results of Mack (2003). While Mack’s data indicated a rise in soil pH, there was no notable distinction between the buttress root zone and the non-buttress root zone (Mack, 2003). During the wet season, the impact of buttress roots on the soil pH value in the 0-10 cm soil layer aligns well with Mack’s findings. A differential nitrogen comparison revealed higher nitrogen content in the basal portion of trees with buttress roots compared to non-buttress root portions. This suggests that buttress roots enhance nitrogen cycling, forming nitrogen reserves and increasing nitrogen availability for plants in tropical rainforests. This finding aligns with the findings of He in the Xishuangbanna region (He, 2012) and further corroborates Pandey et al.’s claim that buttress roots enhance the efficiency of nitrogen element consumption in tropical rainforests (Pandey et al., 2011).

The findings of an increased quantity of organic carbon in the soil of tropical forests through buttress roots align with the study conducted by Dean on eucalyptus trees in Australia (Dean et al., 2020). The relationship analysis of the soil’s primary chemical characteristics revealed a strong link between the total phosphorus element and several other soil indicators in the wet season in the buttress root zone. During the rainy season, there is a strong association between the phosphorus content and organic carbon, nitrogen, and available potassium in the root zone. However, there is a weak correlation with potassium, nitrogen, and phosphorus. Buttress roots contribute to the increase of both the overall amount and efficient use of phosphorus in the soil (Pacaldo and Aydin, 2023). In phosphorus-deficient tropical soils, buttress roots provide a growth advantage, potentially explaining the large stature of these trees (Herwitz, 1988; Pacaldo et al., 2013). In general, buttress roots exert a substantial positive influence on the three primary elements in soil, except potassium.

## 5 Conclusions

In conclusion, buttress roots are essential components in evaluating carbon stocks in tropical forests. Their significant contribution to the amount of carbon in the soil, as well as their diverse ecological functions, highlight their critical role in assessing carbon stocks and developing strategies for managing forests. Our findings revealed that 69.57% of the trees sampled had 3 to 5 buttress roots per tree. The buttress root biomass accounted for 16.18% of the total tree biomass. The total biomass of the buttress roots was calculated to be 8.5 tonnes/ha and 10.7 tonnes/ha, respectively. During both the rainy and dry seasons, it was observed that the presence of buttress roots corresponded to a higher soil organic carbon content and heavy fraction organic carbon by an average of 20.8% in the upslope areas with buttress roots. Recognizing and integrating the impact of buttress roots in our approaches not only improves our understanding of carbon dynamics in tropical forests but also strengthens their ability to act as significant carbon sinks while promoting sustainable forest management practices that conserve biodiversity.

Overall, this study filled an important gap in research and showed that buttress roots can substantially influence carbon dynamics. However, the limitation of the study is the relatively short study period, and therefore, more data collection is essential in unraveling the long-term role that buttress roots play in carbon dynamics mechanisms in tropical forests.

Furthermore, this study offers a foundation for forest managers to recognize and safeguard trees with buttress roots as a silvicultural tactic for carbon forestry and enhancing overall forest well-being. Special conservation attention should be given to trees with buttress roots and sections of the forest where these trees inhabit because of their important ecological benefits such as SOC, soil respiration, and soil nutrient components. These combined endeavors are crucial to the broader goals of mitigating climate change, preserving global biodiversity, and ensuring the long-term sustainability of our planet.

## Data Availability

The raw data supporting the conclusions of this article will be made available by the authors, without undue reservation.
